# Novel five nucleotide deletion in dysferlin leads to autosomal recessive limb‐girdle muscular dystrophy

**DOI:** 10.14814/phy2.15887

**Published:** 2023-12-18

**Authors:** Yen‐Lin Chen, Wen‐Bin Wu, Pei Wang, Ping‐Keung Yip, Yi‐No Wu, Ying‐Hung Lin, Wei‐Ning Lin

**Affiliations:** ^1^ Center for Precision Medicine and Genomics, Tri‐Service General Hospital Medical Defense Medical Center Taipei Taiwan; ^2^ Department of Pathology, Tri‐Service General Hospital Medical Defense Medical Center Taipei Taiwan; ^3^ School of Medicine, College of Medicine Fu Je Catholic University New Taipei City Taiwan; ^4^ Division of Neurology Cardinal Tien Hospital New Taipei City Taiwan; ^5^ Graduate Institute of Biomedical and Pharmaceutical Science Fu Jen Catholic University New Taipei City Taiwan

**Keywords:** dysferlin, heterozygous mutation, limb‐girdle muscular dystrophy

## Abstract

Muscular dystrophy (MD) is a genetic disorder that causes progressive muscle weakness and degeneration. Limb‐girdle muscular dystrophy (LGMD) is a type of MD that mainly causes muscle atrophy within the shoulder and pelvic girdles. LGMD is classified into autosomal dominant (LGMD‐D) and autosomal recessive (LGMD‐R) inheritance patterns. Mutations in the *Dysferlin* gene (*DYSF*) are common causes of LGMD‐R. However, genetic screening of *DYSF* mutations is rare in Taiwan. Herein, we identified a novel c.2867_2871del ACCAG deletion and a previously reported c.937+1G>A mutation in *DYSF* from a Taiwanese family with LGMD. The primary symptoms of both siblings were difficulty climbing stairs, walking on the toes, and gradually worsening weakness in the proximal muscles and increased creatine kinase level. Through pedigree analysis and sequencing, two siblings from this family were found to have compound heterozygous *DYSF* mutations (c. 937+1G>A and c. 2867_2871del ACCAG) within the separated alleles. These mutations induced early stop codons; if translated, truncated DYSF proteins will be expressed. Or, the mRNA products of these two mutations will merit the nonsense‐mediated decay, might result in no dysferlin protein expressed. To our knowledge, this is the first report of a novel c.2867_2871del ACCAG deletion in *DYSF*. Further research is required to examine the effects of the novel *DYSF* mutation in Taiwanese patients with LGMD.

## 1 INTRODUCTION

Muscular dystrophy (MD) is an inherited genetic disorder with muscle weakness and degeneration (Mercuri et al., 2019). Limb‐girdle muscular dystrophy (LGMD) is a type of MD characterized by progressive voluntary muscle atrophy and hip and shoulder faintness, and may affect other body muscles. LGMD is classified as autosomal dominant (LGMD‐D) and autosomal recessive (LGMD‐R) inherited forms (Li et al., 2022). Over 30 genes linked to various identified LGMD subtypes, most of which belong to LGMD‐R. *Dysferlin* (*DYSF* gene), located on chromosome 2p13.3, contains 55 exons and encodes dysferlin with a molecular weight of 230 kDa (Izumi et al., 2015). Mutations in the *DYSF* lead to LGMD‐R2, which belong a type of dysferlinopathy. Dysferlin is a Type II transmembrane protein with an intracellular cytoplasm N‐terminal domain and a extracellular space C‐terminal domain (Kerr et al., 2013). Dysferlin contains seven conserved C2 domains (C2A to C2G) that may interact with calcium, phospholipids, and other proteins (Davis et al., 2002). In addition, dysferlin contains three Fer domains (FerA, FerB, and FerI), two DysF domains (DysFN and DysFC), and an intracellular cytoplasmic N‐terminal domain (Spadafora et al., 2022). Dysferlin is broadly expressed in tissues, especially high expression in skeletal and cardiac muscles (Bashir et al., 1998; Huang et al., 2007; Roche et al., 2008; Salani et al., 2004; Xu et al., 2011; Zhao et al., 2011). Besides, DYSF is associated with the developing T‐tubule system in human skeletal muscles (Klinge et al., 2010).

Recently, the Universal Mutation Database for Dysferlin, a locus‐specific database developed with the UMD® software, has been compiled to provide up‐to‐date information about mutations of the *DYSF* gene (Blandin et al., 2012). It aims to provide accessible information and easy ways for researchers to report their findings. Numerous DYSF mutations have recently been identified in LGMD and classified as LGMD‐R2 (dysferlinopathy) (Millay et al., 2009; Saito et al., 2002; Santos et al., 2010). For example, in Japan, the c.2997G>T mutation in *DYSF* was associated with late‐onset, proximal dominant forms of dysferlinopathy (Takahashi et al., 2013). Recently, more comprehensive genetic screening, and the diagnostic methods of MD have improved in Taiwan (Liang et al., 2020; Lin et al., 2021). However, *DYSF* mutation‐related LGMD data are still rare in Taiwan, resulting in a lack of practical clinical approaches for diagnosing LGMD.

In this study, we identified a novel c.2867_2871del ACCAG deletion in *DYSF* in two siblings from a family with LGMD, which was combined with a c.937+1G>A mutation in the other allele. Family inheritance patterns were revealed, and the c.2867_2871del ACCAG deletion and c.937+1G>A mutation in *DYSF* induced early stop codons which may generate the truncated DYSF proteins, if translated. Or, the mRNA products of these two mutations could cause the nonsense mediated decay, resulting in no dysferlin expressed. To our knowledge, this is the first report of a novel c.2867_2871del ACCAG deletion in *DYSF*.

## 2 MATERIALS AND METHODS

### 2.1 Patient description

**FIGURE 1:**
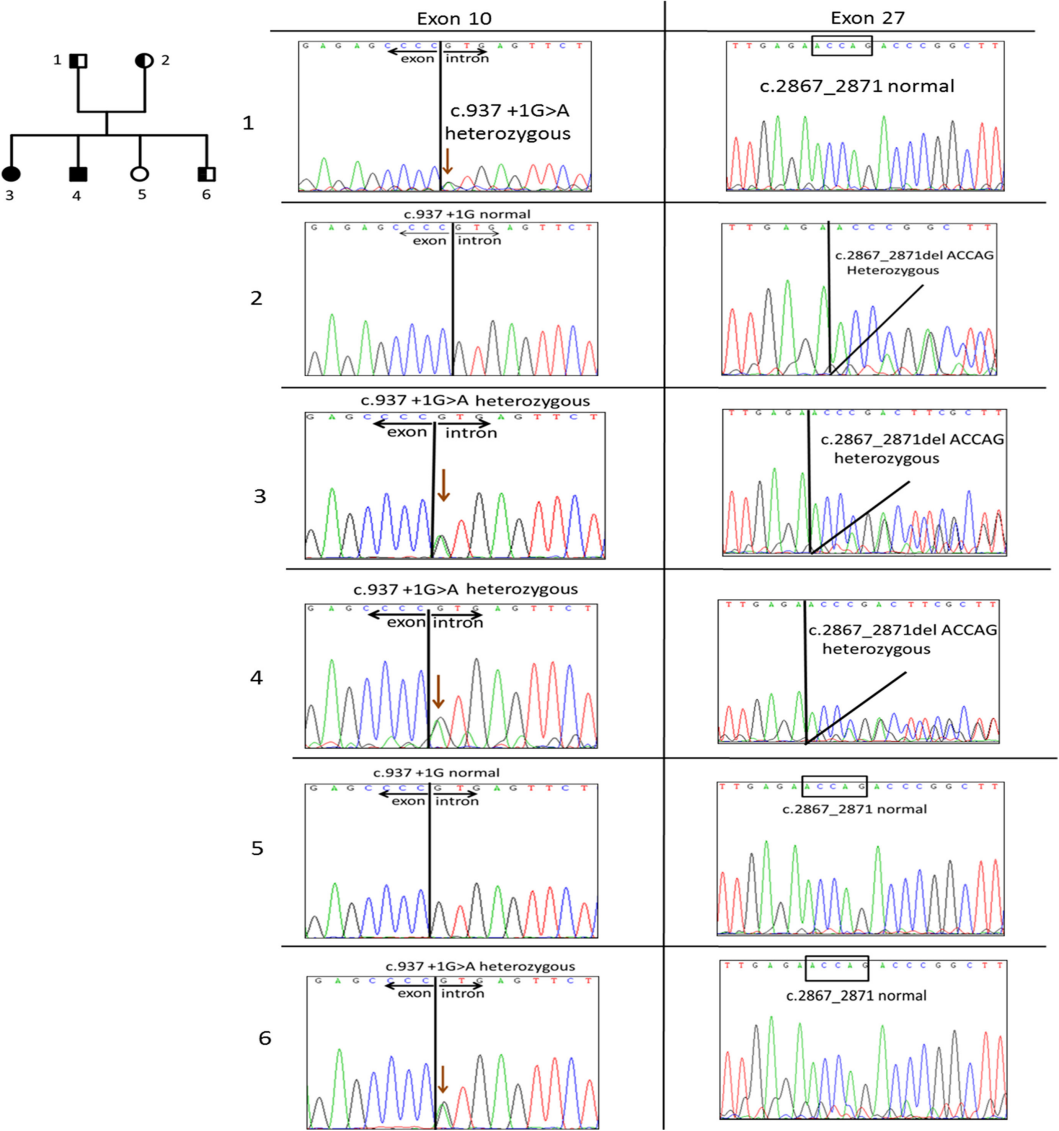
Pedigree and Sanger sequencing analysis of the family with LGMD. Pedigree indicating the affected and healthy siblings (numbers 3–6) and her parents (numbers 1–2) (left panel). Sanger sequencing revealed DYSF mutation sites (right panel). Number 1: c.937 + 1G > A (heterozygous); number 2: c.2867_2871 del ACCAG (heterozygous); numbers 3 and 4: c.937 + 1G > A (heterozygous) and c.2867_2871del ACCAG (heterozygous); number 5: without *DYSF* mutation; and number 6: c.937 + 1G > A (heterozygous).

The study had been reviewed and approved by the Research Ethics Review Committee of the Cardinal Tien Hospital for all bio‐clinical specimens (CTH‐3‐5‐0332021/10/30). There are six members in the LGMD family (refer to Figure 1) were enrolled in this study and the written informed consent was obtained from all patients. Numbered 4 patient were the onset of the disease at the age of 14, gradually, and is 41 years old, now. The primary symptoms were experience of difficulties climbing stairs, walk on the toe and gradually worsening weakness in the proximal muscles, particularly in the calf and thigh. The serum lactate dehydrogenase (LDH) and creatine kinase (CK) were evaluated at diagnosis. Besides, electromyography, cardiology and respiratory tests have analyzed.

### 2.2 DNA preparation, whole‐exon sequence, and data analysis

Blood samples were collected from the subjects (Figure 1), and DNA was isolated using a blood DNA extraction kit (Cat. 51104) (Qiagen, the Netherlands), following the manufacturer's standard protocol. The DNA samples from the proband underwent whole‐exon sequencing, whereby the genomic DNA was enriched using an Agilent SureSelect Human All Exon Enrichment Kit V6 array (Cat. 5190) (Agilent Technologies, Santa Clara, CA, USA), and the fragmented DNA was enhanced and subjected to sequencing using an Illumina NextSeq550 system (Illumina, San Diego, CA, USA). The sequencing depth for each participant was >100‐fold. The NextGENe software was used for alignment, variant calling, and filtering (SOFTGENETICS, State College, PA, USA).

### 2.3 RNA purification, reverse transcription, and polymerase chain reaction (PCR)

RNA was extracted from blood using a Fresh Whole Blood RNA kit (Cat. K0871) (Thermo Fisher Scientific, Waltham, MA, USA). Total RNA (2 μg) was reverse transcribed using the Maxima First Strand cDNA Synthesis Kit (Cat. R1362) (Thermo Fisher Scientific, Waltham, MA, USA) for RT‐PCR. Both kits were used according to the manufacturer's instructions. For PCR, dysferlin primers 5′‐TCGTTCTCTCAGGACAGATGC‐3′ (sense) and 5′‐CTGAGGGTTGGCCGTC TT‐3′ (antisense) were used. The cDNA samples were amplified using PCR using Hotstart master mix DNA polymerase (Cat. M0494) (New England Biolabs, Ipswich, MA, USA) as follows: 98°C for 5 min, 30 cycles of 98°C for 30 s, 59.1°C for 30 s, 68°C for 1 min, and then 68°C for 7 min. PCR products were analyzed using gel electrophoresis. PCR products were subjected to Sanger sequencing.

### 2.4 Bioinformation evaluation and three‐dimensional (3D) structure prediction

To predict the translated amino acid sequence or molecular weight of DYSF with c.937+1G>A or c.2867_2871del ACCAG mutations, the ExPASy Translate server (http://web.expasy.org/translate/) together with ExPASy Compute pI/Mw (https://web.expasy.org/compute_pi/) was used according to the manufacturer's instructions. An artificial intelligence system, AlphaFold, developed by DeepMind, was used to predict the 3D structure of mutated DYSF amino acid sequences (Spadafora et al., 2022). The 3D structural models of DYSF were coded into the wild‐type file O75923 model.

## 3 RESULTS

### 3.1 Identified of a novel c.2867_2871del ACCAG deletion in *DYSF* in LGMD patients

First, the proband (Number 4; Figure 1), currently 41 years old, experienced disease onset at the age of 14 years. The primary symptoms were difficulty climbing stairs, walking on the toes, and gradually worsening weakness of the proximal muscles, particularly the calf and thigh. Serum lactate dehydrogenase and creatine kinase of the patient were elevated as 325 and 2484 U/L, respectively. And, no any abnormalities were found within the cardiological and respiratory tests. Following, the patient was diagnosed with muscular dystrophy. To explore the possible genetic mutation in the patient (number 4), whole exons sequencing was applied. *DYSF* mutations, c.937+1G>A (exon 10) and c.2867_2871del ACCAG (exon 27), were identified. To track the inheritance of this family, the exons 10 and 27 of *DYSF* were sequenced in the other family members (Figure 1). The inheritance patterns of the two *DYSF* mutations in the family are shown in Figure 1 (right panel). Among the siblings, both types of mutations (c.937+1G>A and c.2867_2871del ACCAG) were present in siblings 3 and 4 (current patient). The younger brother was a carrier of a single c.937+1G>A mutation (number 6), and the younger sister did not exhibit either of the two mutation phenotypes (number 5). The father and mother carried the c.937+1G>A and c.2867_2871del ACCAG mutations, respectively. Herein, we identified a novel deletion, c.2867_2871del ACCAG, and a previously described c.937+1G>A mutation in *DYSF* in a family member with LGMD.

### 3.2 The c.937+1G>A mutation in *DYSF* activate a cryptic splice donor site

**FIGURE 2:**
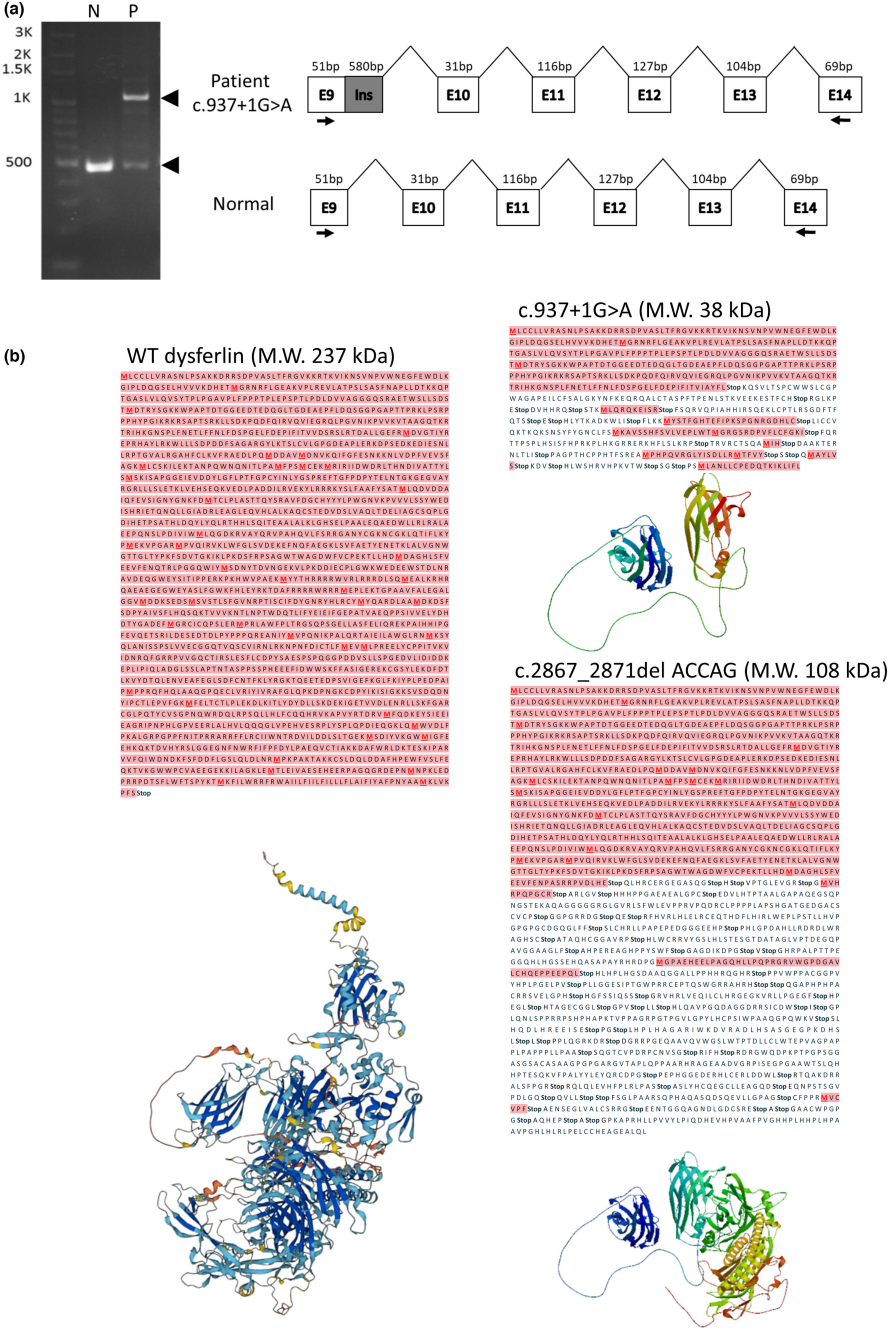
Effects of *DYSF* c.937 + 1G > A mutation on the splicing process. (a) Graphical presentation of the wild‐type and aberrantly spliced transcripts using RT‐PCR from normal (N) and *DYSF* c.937 + 1G > A patient (P; number 6) left panel). The *DYSF* c.937 + 1G > A mutation induced alternative splicing in vivo (right panel). RT‐PCR fragments containing exons 10–14 (498 bp), and alternative processes induced alternative splicing (1078 bp), which resulted in the insertion of 580 bp (partial intron 10), shown as a gray box. (b) Prediction of mutant and full length DYSF translation. The translated amino acid of DYSF with wild type (left), c.937 + 1G > A (upper right), and c.2867_2871del ACCAG (lower right) mutation sequences were obtained by the ExPASy Translate server (https://web.expasy.org/translate/) and the potent protein models were predicted by the WEISS‐MODEL with the wild type template of dysferlin (O75923).

In a previous study, the c.937+1G>A mutation in *DYSF* was suggested to be pathogenic through alternative splicing. To evaluate the hypothesis, the RNA transcripts were isolated from blood and subjected to RT‐PCR analysis in Figure 2a (left panel). The c.937+1G>A disrupts the splicing donor site between exon 9 and exon 10 and induces a cryptic splicing donor site in intron 9 in Figure 2a (right panel). Abnormal splicing results in the abnormal transcripts (the size of PCR product: 1078 bp), which inserts 580 bp from intron 9 in patients with the c.937+1G>A mutation, compared to the 498 bp DNA fragment in control. The novel transcript therefore encodes a truncated protein lacking the large part of c‐terminal *DYSF*. Regarding the new c.2867_2871del ACCAG deletion, RT‐PCR is currently less sensitive in detecting a 5‐nucleotide deletion, and mice with the c.2867_2871del ACCAG deletion are in the process of being designed. To determine whether DYSF mutations affect its protein if translation, ExPASy prediction model was applied. The expression of wild type DYSF gene resulted in a 237 kDa protein (Figure 2b left panel). Translation prediction revealed, that the RNA transcript resulting from abnormal splicing, was translated into a small truncated DYSF protein with predicted M.W. 38 kDa (Figure 2b upper right panel). By contrast, c.2867_2871del ACCAG is a novel deletion that has not been reported in the literature, international dysferlin database, or other genetic disease databases. Base on the deleted five nucleotides, a frameshift was logically assumed to induce an early stop codon during translation. An early stop codon was presumed using translation prediction software, resulting in a truncated DYSF protein (predicted M.W. 108 kDa) (Figure 2b lower right panel).

## 4 DISCUSSION

*DYSF* shares sequence homology with fer‐1, which mediates vesicle fusion with the plasma membrane in spermatids of *Caenorhabditis elegans* (Achanzar & Ward, 1997). In normal muscles, DYSF proteins are enriched in membrane patches and respond to sarcolemma injury (Covian‐Nares et al., 2010). When membrane disruption occurs, a rapid Ca^2+^ flood triggers the aggregation of DYSF‐carrying repair vesicles and transports them toward the disruption site to reseal the membrane (Azakir et al., 2010). By contrast, abnormal accumulation of vesicles within sub‐sarcolemmal region is in the *Dysferlin*‐null muscle, due to lack of precise vesicle regulation (Bansal et al., 2003). A recent study also showed that DYSF can stabilize stress‐induced Ca^2+^ signaling in the transverse tubule membrane (Han & Campbell, 2007) and regulate Ca^2+^ homeostasis in the skeletal muscle (Kerr et al., 2014). In this study, we identified c.2867_2871del ACCAG deletion and c.937+1G>A mutation of *DYSF*, which were existed in separated alleles in the patient. The c.937+1G>A mutation in *DYSF* is predicted to generate a premature stop codon that could result in nonsense‐mediated decay of the transcript with no dysferlin protein expressed. If translated, the shorter open reading frame is predicted to generate a truncated form (38 kDa) of dysferlin. Another novel c.2867_2871del ACCAG deletion of *DYSF* may create a frameshift in the transcript, which possible also merit the process of nonsense‐mediated decay, and resulting in no dysferlin expressed. Alternatively, this deletion might also result in the expression of a truncated 108 kDa protein if translated. Briefly, the mRNA products of these two mutations, c.937+1G>A or c.2867_2871del ACCAG, might translate and generate the truncated dysferlin protein with 38 kDa or 108 kDa separately if the nonsense‐mediated mRNA decay is not operative. Compared to the wild‐type dysferlin protein (237 kDa), only the N‐terminal portion of the truncated dysferlin protein might be expressed. In addition, the mRNA expression of the c.937+1G>A mutation seems to possibly be higher than that of the c.2867_2871del ACCAG deletion. However, the precise mRNA contents or even protein abundances of the c.937+1G>A mutation and c.2867_2871del ACCAG deletion needs to be evaluated in the future. Mice with c.2867_2871del ACCAG deletion or mice with two alleles carrying either the c.937+1G>A or the c.2867_2871del ACCAG mutation are in the processes. The precise molecular defects form underlying c.937+1G>A and c.2867_2871del ACCAG mutations remain unidentified and will be evaluated in the future.

Past decade, α‐tubulin was identified as a novel DYSF binding partner using affinity purification followed by liquid chromatography/mass spectrometry (Azakir et al., 2010; Demonbreun et al., 2014). The cellular function of microtubules, composed of α and β tubulin, can be regulated by posttranslational modifications. A recent study showed that DYSF interacts with histone deacetylase 6 through the C2D domain, preventing α‐tubulin deacetylation in cos‐7 cells and myoblasts. In addition, increased levels of α‐tubulin acetylation in DYSF‐expressing cells and myoblasts may enhance myotube formation (Balasubramanian et al., 2014). How mutations c.937+1G>A and c.2867_2871del ACCAG damage the construction of myotube during myogenesis remains to be evaluated.

Furthermore, RT‐PCR and Northern blot analyses revealed that *DYSF* mRNA was also expressed in endothelial cells such as the bovine aorta and human umbilical vein endothelial cells. Knockdown of dysferlin using siRNA in subconfluent endothelial cells caused deficient adhesion, suggesting the possibility of DYSF in angiogenesis (Sharma et al., 2010). A recent study found that the loss of *DYSF* in human monocytes strongly reduced adhesion and increased motility, which might influence the infiltration of dysferlinopathy muscles (de Morrée et al., 2013). Hence, DYSF may modulate the adhesion of inflammatory cells and even angiogenesis during muscle repair. Whether the c.937+1G>A or c.2867_2871del ACCAG mutations facilitate the infiltration and accumulation of inflammatory cells and delay tissue repair in myotubes remains to be clarified.

## 5 CONCLUSIONS

Many patients harboring *DYSF* gene mutations have been reported. In this study, we focus on identification of *DYSF* mutations in Taiwanese cases with LGMD. Specifically, we identified a pathogenic c.2867_2871del ACCAG of DYSF mutation that first reported here. Further understanding the molecular effects of *DYSF* mutation sites will be addressed in the focus on modulating inflammation together with skeletal muscle repair, following this study.

## AUTHOR CONTRIBUTIONS

Yen‐Lin Chen, Yi‐No Wu, Ying‐Hung Lin, and Wen‐Bin Wu conceived and designed the study; Yen‐Lin Chen performed experiments and analyzed the data; Yen‐Lin Chen, Wen‐Bin Wu, Pei Wang, Ping‐Keung Yip, Yi‐No Wu, Ying‐Hung Lin, and Wen‐Bin Wu interpreted the experimental results; Yen‐Lin Chen Yi‐No Wu, Ying‐Hung Lin, and Wen‐Bin Wu prepared the figures; Yen‐Lin Chen and Wen‐Bin Wu drafted the manuscript; Yi‐No Wu and Ying‐Hung Lin edited and revised the manuscript.

## CONFLICT OF INTEREST STATEMENT

The authors declare no conflicts of interest, financial or otherwise.

## ETHICS APPROVAL AND CONSENT TO PARTICIPATE

The studies involving human participants were reviewed and approved by the Research Ethics Review Committee of the Cardinal Tien Hospital for all bio‐clinical specimens (**CTH‐3‐5‐0332021/10/30**). All patients provided informed written consent to participate in the project.

## CONSENT FOR PUBLICATION

Written informed consent was obtained from the patients for publication.

## Data Availability

The data generated from the whole‐exon sequence is available from the corresponding author on reasonable request.

## References

[phy215887-bib-0001] Achanzar, W. E. , & Ward, S. (1997). A nematode gene required for sperm vesicle fusion. Journal of Cell Science, 110(Pt 9), 1073–1081. 9175703 10.1242/jcs.110.9.1073

[phy215887-bib-0002] Azakir, B. A. , Di Fulvio, S. , Therrien, C. , & Sinnreich, M. (2010). Dysferlin interacts with tubulin and microtubules in mouse skeletal muscle. PLoS One, 5(4), e10122. 20405035 10.1371/journal.pone.0010122 PMC2853571

[phy215887-bib-0003] Balasubramanian, A. , Kawahara, G. , Gupta, V. A. , Rozkalne, A. , Beauvais, A. , Kunkel, L. M. , & Gussoni, E. (2014). Fam65b is important for formation of the HDAC6‐dysferlin protein complex during myogenic cell differentiation. FASEB Journal: Official Publication of the Federation of American Societies for Experimental Biology, 28(7), 2955–2969. 24687993 10.1096/fj.13-246470 PMC4062822

[phy215887-bib-0004] Bansal, D. , Miyake, K. , Vogel, S. S. , Groh, S. , Chen, C. C. , Williamson, R. , McNeil, P. L. , & Campbell, K. P. (2003). Defective membrane repair in dysferlin‐deficient muscular dystrophy. Nature, 423(6936), 168–172. 12736685 10.1038/nature01573

[phy215887-bib-0005] Bashir, R. , Britton, S. , Strachan, T. , Keers, S. , Vafiadaki, E. , Lako, M. , Richard, I. , Marchand, S. , Bourg, N. , Argov, Z. , Sadeh, M. , Mahjneh, I. , Marconi, G. , Passos‐Bueno, M. R. , Moreira Ede, S. , Zatz, M. , Beckmann, J. S. , & Bushby, K. (1998). A gene related to Caenorhabditis elegans spermatogenesis factor fer‐1 is mutated in limb‐girdle muscular dystrophy type 2B. Nature Genetics, 20(1), 37–42. 9731527 10.1038/1689

[phy215887-bib-0006] Blandin, G. , Beroud, C. , Labelle, V. , Nguyen, K. , Wein, N. , Hamroun, D. , Williams, B. , Monnier, N. , Rufibach, L. E. , Urtizberea, J. A. , Cau, P. , Bartoli, M. , Lévy, N. , & Krahn, M. (2012). UMD‐DYSF, a novel locus specific database for the compilation and interactive analysis of mutations in the dysferlin gene. Human Mutation, 33(3), E2317–E2331. 22213072 10.1002/humu.22015

[phy215887-bib-0007] Covian‐Nares, J. F. , Koushik, S. V. , Puhl, H. L., 3rd , & Vogel, S. S. (2010). Membrane wounding triggers ATP release and dysferlin‐mediated intercellular calcium signaling. Journal of Cell Science, 123(Pt 11), 1884–1893. 20442251 10.1242/jcs.066084 PMC2873225

[phy215887-bib-0008] Davis, D. B. , Doherty, K. R. , Delmonte, A. J. , & McNally, E. M. (2002). Calcium‐sensitive phospholipid binding properties of normal and mutant ferlin C2 domains. The Journal of Biological Chemistry, 277(25), 22883–22888. 11959863 10.1074/jbc.M201858200

[phy215887-bib-0009] de Morrée, A. , Flix, B. , Bagaric, I. , Wang, J. , van den Boogaard, M. , Grand Moursel, L. , Frants, R. R. , Illa, I. , Gallardo, E. , Toes, R. , & van der Maarel, S. M. (2013). Dysferlin regulates cell adhesion in human monocytes. The Journal of Biological Chemistry, 288(20), 14147–14157. 23558685 10.1074/jbc.M112.448589 PMC3656271

[phy215887-bib-0010] Demonbreun, A. R. , Rossi, A. E. , Alvarez, M. G. , Swanson, K. E. , Deveaux, H. K. , Earley, J. U. , Hadhazy, M. , Vohra, R. , Walter, G. A. , Pytel, P. , & McNally, E. M. (2014). Dysferlin and myoferlin regulate transverse tubule formation and glycerol sensitivity. The American Journal of Pathology, 184(1), 248–259. 24177035 10.1016/j.ajpath.2013.09.009 PMC3873498

[phy215887-bib-0011] Han, R. , & Campbell, K. P. (2007). Dysferlin and muscle membrane repair. Current Opinion in Cell Biology, 19(4), 409–416. 17662592 10.1016/j.ceb.2007.07.001 PMC2144911

[phy215887-bib-0012] Huang, Y. , Laval, S. H. , van Remoortere, A. , Baudier, J. , Benaud, C. , Anderson, L. V. , Straub, V. , Deelder, A. , Frants, R. R. , den Dunnen, J. T. , Bushby, K. , & van der Maarel, S. M. (2007). AHNAK, a novel component of the dysferlin protein complex, redistributes to the cytoplasm with dysferlin during skeletal muscle regeneration. FASEB journal: official publication of the Federation of American Societies for Experimental Biology, 21(3), 732–742. 17185750 10.1096/fj.06-6628com

[phy215887-bib-0013] Izumi, R. , Niihori, T. , Takahashi, T. , Suzuki, N. , Tateyama, M. , Watanabe, C. , Sugie, K. , Nakanishi, H. , Sobue, G. , Kato, M. , Warita, H. , Aoki, Y. , & Aoki, M. (2015). Genetic profile for suspected dysferlinopathy identified by targeted next‐generation sequencing. Neurol Genet, 1(4), e36. 27066573 10.1212/NXG.0000000000000036 PMC4811388

[phy215887-bib-0014] Kerr, J. P. , Ward, C. W. , & Bloch, R. J. (2014). Dysferlin at transverse tubules regulates Ca(2^+^) homeostasis in skeletal muscle. Frontiers in Physiology, 5, 89. 24639655 10.3389/fphys.2014.00089 PMC3944681

[phy215887-bib-0015] Kerr, J. P. , Ziman, A. P. , Mueller, A. L. , Muriel, J. M. , Kleinhans‐Welte, E. , Gumerson, J. D. , Vogel, S. S. , Ward, C. W. , Roche, J. A. , & Bloch, R. J. (2013). Dysferlin stabilizes stress‐induced Ca2+ signaling in the transverse tubule membrane. Proceedings of the National Academy of Sciences of the United States of America, 110(51), 20831–20836. 24302765 10.1073/pnas.1307960110 PMC3870721

[phy215887-bib-0016] Klinge, L. , Harris, J. , Sewry, C. , Charlton, R. , Anderson, L. , Laval, S. , Chiu, Y. H. , Hornsey, M. , Straub, V. , Barresi, R. , Lochmüller, H. , & Bushby, K. (2010). Dysferlin associates with the developing T‐tubule system in rodent and human skeletal muscle. Muscle & Nerve, 41(2), 166–173. 20082313 10.1002/mus.21166

[phy215887-bib-0017] Li, C. , Haller, G. , & Weihl, C. C. (2022). Current and future approaches to classify VUSs in LGMD‐related genes. Genes, 13(2), 382. 35205425 10.3390/genes13020382 PMC8871643

[phy215887-bib-0018] Liang, W. C. , Jong, Y. J. , Wang, C. H. , Wang, C. H. , Tian, X. , Chen, W. Z. , Kan, T. M. , Minami, N. , Nishino, I. , & Wong, L. C. (2020). Clinical, pathological, imaging, and genetic characterization in a Taiwanese cohort with limb‐girdle muscular dystrophy. Orphanet J Rare Dis, 15(1), 160. 32576226 10.1186/s13023-020-01445-1 PMC7310488

[phy215887-bib-0019] Lin, C. W. , Tsui, P. H. , Lu, C. H. , Hung, Y. H. , Tsai, M. R. , Shieh, J. Y. , & Weng, W. C. (2021). Quantifying Lower Limb Muscle Stiffness as Ambulation Function Declines in Duchenne Muscular Dystrophy with Acoustic Radiation Force Impulse Shear Wave Elastography. Ultrasound in Medicine & Biology, 47(10), 2880–2889. 34284931 10.1016/j.ultrasmedbio.2021.06.008

[phy215887-bib-0020] Mercuri, E. , Bönnemann, C. G. , & Muntoni, F. (2019). Muscular dystrophies. Lancet (London, England), 394(10213), 2025–2038. 31789220 10.1016/S0140-6736(19)32910-1

[phy215887-bib-0021] Millay, D. P. , Maillet, M. , Roche, J. A. , Sargent, M. A. , McNally, E. M. , Bloch, R. J. , & Molkentin, J. D. (2009). Genetic manipulation of dysferlin expression in skeletal muscle: novel insights into muscular dystrophy. The American Journal of Pathology, 175(5), 1817–1823. 19834057 10.2353/ajpath.2009.090107 PMC2774048

[phy215887-bib-0022] Roche, J. A. , Lovering, R. M. , & Bloch, R. J. (2008). Impaired recovery of dysferlin‐null skeletal muscle after contraction‐induced injury in vivo. Neuroreport, 19(16), 1579–1584. 18815587 10.1097/WNR.0b013e328311ca35 PMC2662728

[phy215887-bib-0023] Saito, A. , Higuchi, I. , Nakagawa, M. , Saito, M. , Hirata, K. , Suehara, M. , Yoshida, Y. , Takahashi, T. , Aoki, M. , & Osame, M. (2002). Miyoshi myopathy patients with novel 5′ splicing donor site mutations showed different dysferlin immunostaining at the sarcolemma. Acta Neuropathologica, 104(6), 615–620. 12410383 10.1007/s00401-002-0593-x

[phy215887-bib-0024] Salani, S. , Lucchiari, S. , Fortunato, F. , Crimi, M. , Corti, S. , Locatelli, F. , Bossolasco, P. , Bresolin, N. , & Comi, G. P. (2004). Developmental and tissue‐specific regulation of a novel dysferlin isoform. Muscle & Nerve, 30(3), 366–374. 15318348 10.1002/mus.20106

[phy215887-bib-0025] Santos, R. , Oliveira, J. , Vieira, E. , Coelho, T. , Carneiro, A. L. , Evangelista, T. , Dias, C. , Fortuna, A. , Geraldo, A. , Negrão, L. , Guimarães, A. , & Bronze‐da‐Rocha, E. (2010). Private dysferlin exon skipping mutation (c.5492G>a) with a founder effect reveals further alternative splicing involving exons 49‐51. Journal of Human Genetics, 55(8), 546–549. 20535123 10.1038/jhg.2010.60

[phy215887-bib-0026] Sharma, A. , Yu, C. , Leung, C. , Trane, A. , Lau, M. , Utokaparch, S. , Shaheen, F. , Sheibani, N. , & Bernatchez, P. (2010). A new role for the muscle repair protein dysferlin in endothelial cell adhesion and angiogenesis. Arteriosclerosis, Thrombosis, and Vascular Biology, 30(11), 2196–2204. 20724702 10.1161/ATVBAHA.110.208108 PMC2996267

[phy215887-bib-0027] Spadafora, P. , Qualtieri, A. , Cavalcanti, F. , Di Palma, G. , Gallo, O. , De Benedittis, S. , Cerantonio, A. , & Citrigno, L. (2022). A novel homozygous variant in DYSF gene is associated with autosomal recessive limb girdle muscular dystrophy R2/2B. International Journal of Molecular Sciences, 23(16), 8932. 36012197 10.3390/ijms23168932 PMC9408934

[phy215887-bib-0028] Takahashi, T. , Aoki, M. , Suzuki, N. , Tateyama, M. , Yaginuma, C. , Sato, H. , Hayasaka, M. , Sugawara, H. , Ito, M. , Abe‐Kondo, E. , Shimakura, N. , Ibi, T. , Kuru, S. , Wakayama, T. , Sobue, G. , Fujii, N. , Saito, T. , Matsumura, T. , Funakawa, I. , … Itoyama, Y. (2013). Clinical features and a mutation with late onset of limb girdle muscular dystrophy 2B. Journal of Neurology, Neurosurgery, and Psychiatry, 84(4), 433–440. 23243261 10.1136/jnnp-2011-301339 PMC3595148

[phy215887-bib-0029] Xu, L. , Pallikkuth, S. , Hou, Z. , Mignery, G. A. , Robia, S. L. , & Han, R. (2011). Dysferlin forms a dimer mediated by the C2 domains and the transmembrane domain in vitro and in living cells. PLoS One, 6(11), e27884. 22110769 10.1371/journal.pone.0027884 PMC3215728

[phy215887-bib-0030] Zhao, P. , Xu, L. , Ait‐Mou, Y. , de Tombe, P. P. , & Han, R. (2011). Equal force recovery in dysferlin‐deficient and wild‐type muscles following saponin exposure. Journal of Biomedicine & Biotechnology, 2011, 235216. 21941430 10.1155/2011/235216 PMC3175419

